# Effects of Patient Characteristics on Diagnostic Performance of Self-Collected Samples for SARS-CoV-2 Testing

**DOI:** 10.3201/eid2708.210667

**Published:** 2021-08

**Authors:** Sarah E. Smith-Jeffcoat, Mitsuki Koh, Adam Hoffman, Paulina A. Rebolledo, Marcos C. Schechter, Halie K. Miller, Sadia Sleweon, Rebecca Rossetti, Vyjayanti Kasinathan, Talya Shragai, Kevin O’Laughlin, Catherine C. Espinosa, George M. Khalil, AdeSubomi O. Adeyemo, Anne Moorman, Brenda L. Bauman, Kahaliah Joseph, Michelle O’Hegarty, Nazia Kamal, Hany Atallah, Brooks L. Moore, Caitlin D. Bohannon, Bettina Bankamp, Claire Hartloge, Michael D. Bowen, Ashley Paulick, Amy S. Gargis, Christopher Elkins, Rebekah J. Stewart, Juliana da Silva, Caitlin Biedron, Jacqueline E. Tate, Yun F. Wang, Hannah L. Kirking, the CDC COVID-9, Response Team

**Affiliations:** Centers for Disease Control and Prevention, Atlanta, Georgia, USA (S.E. Smith-Jeffcoat, M. Koh, H.K. Miller, S. Sleweon, R. Rossetti, T. Shragai, K. O’Laughlin, C.C. Espinosa, G.M. Khalil, A.O. Adeyemo, A. Moorman, B.L. Bauman, K. Joseph, M. O’Hegarty, N. Kamal, C.D. Bohannon, B. Bankamp, C. Hartlodge, M.D. Bowen, A. Paulick, A.S. Gargis, C. Elkins, R.J. Stewart, J. da Silva, C. Biedron, J.E. Tate, H.L. Kirking);; Public Health Institute—CDC Global Health Fellowship Program, Atlanta (M. Koh);; Emory University, Atlanta (A. Hoffman, P.A. Rebolledo, M.C. Schechter, V. Kasinathan, H. Atallah, B.L. Moore, Y.F. Wang);; Grady Memorial Hospital, Atlanta (A. Hoffman, P.A. Rebolledo, M.C. Schechter, V. Kasinathan, H. Atallah, B.L. Moore, Y.F. Wang)

**Keywords:** self-collected, saliva, anterior nasal, SARS-CoV-2, sensitivity, respiratory infections, severe acute respiratory syndrome coronavirus 2, 2019 novel coronavirus disease, coronavirus disease, zoonoses, viruses, coronaviruses, COVID-19, Georgia, United States

## Abstract

We evaluated the performance of self-collected anterior nasal swab (ANS) and saliva samples compared with healthcare worker–collected nasopharyngeal swab specimens used to test for severe acute respiratory syndrome coronavirus 2 (SARS-CoV-2). We used the same PCR diagnostic panel to test all self-collected and healthcare worker–collected samples from participants at a public hospital in Atlanta, Georgia, USA. Among 1,076 participants, 51.9% were men, 57.1% were >50 years of age, 81.2% were Black (non-Hispanic), and 74.9% reported >1 chronic medical condition. In total, 8.0% tested positive for SARS-CoV-2. Compared with nasopharyngeal swab samples, ANS samples had a sensitivity of 59% and saliva samples a sensitivity of 68%. Among participants tested 3–7 days after symptom onset, ANS samples had a sensitivity of 80% and saliva samples a sensitivity of 85%. Sensitivity varied by specimen type and patient characteristics. These findings can help physicians interpret PCR results for SARS-CoV-2.

Detection of severe acute respiratory syndrome coronavirus 2 (SARS-CoV-2), the virus that causes coronavirus disease (COVID-19), originally relied mainly on nasopharyngeal swab (NPS) samples collected by healthcare workers (HCWs). However, NPS sample collection requires substantial amounts of time and personal protective equipment (PPE) that could be preferentially used for patient care. In light of >98 million confirmed COVID-19 cases globally as of January 27, 2021, relying solely on HCW-collected specimens for testing is not feasible (*1*). During the COVID-19 pandemic, many healthcare sites have experienced shortages of PPE and testing supplies. In addition, NPS sample collection often causes coughing or sneezing, which can generate infectious aerosols and thereby put the HCW at increased risk for exposure (*2*). Furthermore, NPS collection can cause discomfort and occasional nosebleeds, possibly affecting a patient’s willingness to be retested. The use of self-collected saliva and anterior nasal swab (ANS) samples reduces HCW contact, limits need for PPE, and preserves transport media and other collection supplies needed for NPS samples.

Various upper respiratory specimen types, including saliva and oral swab samples, have demonstrated similar sensitivity to NPS samples in nucleic acid amplification tests for SARS-CoV-2 (*3*–*6*). However, most patients in these studies reported the recent onset of respiratory symptoms. Other investigations have shown that many infected persons, especially those who are young and otherwise healthy, are asymptomatic or have mild symptoms (*7*–*9*). SARS-CoV-2 RNA has been detected in NPS samples nearly 2 months after initial detection; however, the performance of self-collected ANS and saliva samples of patients with prolonged viral shedding remains unclear (*10*,*11*). Understanding how these less invasive, self-collected specimens perform in a variety of contexts can inform testing strategies. We compared the diagnostic performance of self-collected ANS and saliva samples and HCW-collected NPS samples used in SARS-CoV-2–specific PCR by patient characteristics and symptom status.

## Methods

We recruited patients from several inpatient and outpatient departments of Grady Memorial Hospital (Atlanta, GA, USA), where a high proportion of patients are uninsured (24%) or have Medicare/Medicaid insurance (57%) (*12*). Patients were eligible if their treating physician ordered collection of an NPS sample for SARS-CoV-2–specific reverse transcription PCR (RT-PCR) for any reason, including diagnostic (e.g., patients were symptomatic or exposed) or screening (e.g., preoperative requirement or before admission for non–COVID-19 reasons) purposes. Patients were excluded if they were unable to provide consent, declined consent, were <18 years of age, had a contraindicated NPS specimen (e.g., had a condition that prevented NPS sample collection), were unable to self-collect specimens, or had previously participated in this investigation. Trained interviewers used a standardized questionnaire to collect data on patient demographics, reason for visit, current and previous symptoms, and medical history (including previous SARS-CoV-2 testing). Each participant received a US $25 gift card.

During interviews, patients were given an infographic outlining steps for self-collection of saliva and ANS samples (Appendix Figure) (*13*). Patients self-collected raw (unenhanced) saliva in a 50-mL tube. Patients then inserted a miniature flocked-tip swab into 1 anterior naris, twirled the swab for 10 seconds, removed the swab and placed it directly into the other naris, and twirled it again for 10 seconds. Patients inserted the swab into 3 mL of viral transport media. After the interview and self-collection of specimens, a HCW collected an NPS sample from the participant and inserted the swab into 3 mL of viral transport media. Hospital laboratory staff conducted RT-PCR on the NPS sample on the same day; these results were used to inform clinical care and were not included in the performance analysis. NPS samples then were aliquoted and transferred to the Centers for Disease Control and Prevention (CDC) for RT-PCR. CDC staff extracted nucleic acid and tested samples using the CDC 2019-nCoV Real-Time Reverse Transcription PCR (rRT-PCR) Diagnostic Panel, which is selective for the SARS-CoV-2 nucleocapsid 1 (N1) and 2 (N2) genes, as per the Emergency Use Authorization Instructions for Use (*14*,*15*) (Appendix).

We entered and stored completed questionnaires and laboratory results in a REDCap version 10.0.8 (https://www.project-redcap.org) database hosted at CDC. We grouped patients according to COVID-19 symptom status: always asymptomatic participants reported no COVID-19 symptoms at specimen collection or in the previous 14 days; currently asymptomatic participants reported no COVID-19 symptoms at specimen collection but had symptoms in the previous 14 days; and currently symptomatic participants reported COVID-19 symptoms at specimen collection. We categorized symptoms according to previously defined case definitions (*16*–*18*) (Appendix). We calculated sample size using a 1-sided, 1-sample proportions test with a continuity correction to determine whether sensitivity of self-collected samples was >90% compared with HCW-collected NPS samples, assuming that NPS samples had a true sensitivity of 98% (*3*). Using α = 0.05, 80% power, and 5% NPS percent positivity, we calculated the minimum sample size to be 920 and the required number of positive self-collected specimens to be 46.

We compiled demographic and clinical characteristics for patients according to the results of their NPS samples. To analyze the benefit of using both self-collected ANS and saliva specimens for diagnosis, we merged each patient’s ANS and swab sample results to create a self-collected combination result. If >1 self-collected specimen was positive, we marked that patient’s self-collected combination result as positive. If neither was positive and >1 was negative, then we marked that patient’s self-collected combination result as negative. We calculated sensitivity, specificity, positive predictive value (PPV), and negative predictive value (NPV) of ANS, saliva, and self-collected combination samples compared with NPS samples for all patients who had a definitive (i.e., positive or negative) NPS result and >1 self-collected specimen. Because NPS samples do not show all SARS-CoV-2 infections, we reran the sensitivity analysis with a combined variable for any positive result from ANS, saliva, or NPS samples as the comparator. We compiled proportions of concordant and discordant results for each self-collected and HCW-collected sample and calculated Cohen’s κ coefficient to compare result agreement by specimen type. We calculated the sensitivity of self-collected specimens by patient characteristics and determined significant differences using a 1-sample, 2-sided test of proportions (p<0.05). We used the Pearson correlation coefficient to compare the cycle threshold (C_t_) values of positive self-collected and HCW-collected specimens; we used the Mann-Whitney U test to compare the C_t_ values of NPS samples by patient characteristic. We analyzed the data in R version 4.0.2 (The R Project for Statistical Computing, https://www.r-project.org).

This investigation was reviewed by CDC and conducted in accordance with applicable federal law and CDC policy (e.g., 45 C.F.R. part 46, 21 C.F.R. part 56, 42 U.S.C.; 241(d); 5 U.S.C. 552a; 44 U.S.C. 3501 et seq.). This investigation was determined to be an exempt public health activity by the Emory University Institutional Review Board and Grady Memorial Hospital Research Oversight Committee.

## Results

**Figure 1:**
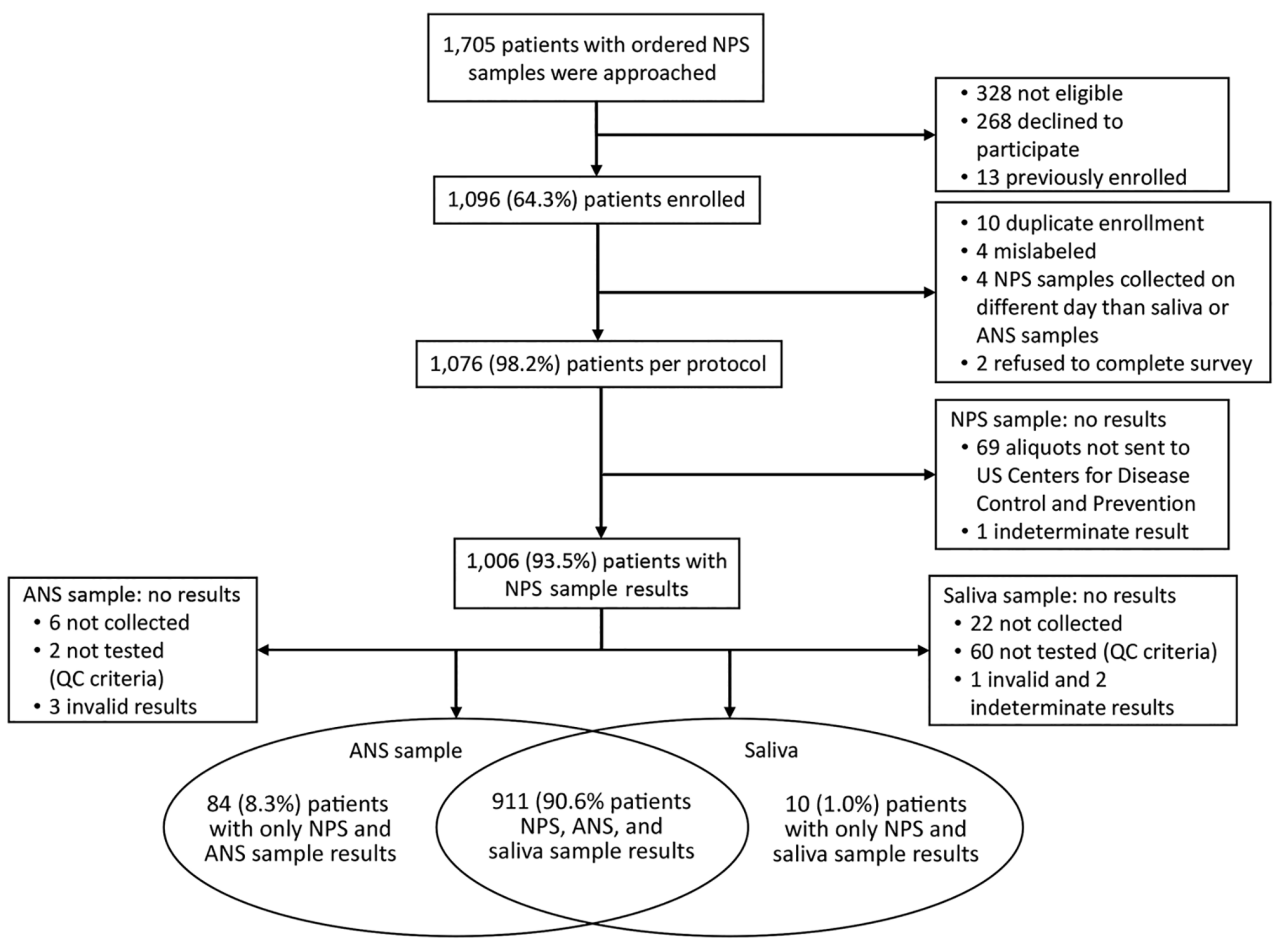
Flowchart of patient enrollment and sample results for investigation of the effects of patient characteristics on self-collected and healthcare worker–collected samples for severe acute respiratory syndrome coronavirus 2 testing, Atlanta, Georgia, USA. ANS, anterior nasal swab; NPS, nasopharyngeal swab; QC, quality control.

During August 31–November 23, 2020, a total of 1,096 patients consented to and enrolled in the study; 20 were excluded because they did not meet inclusion criteria (Figure 1). Among 1,076 participants, overall positivity of any specimen was 8.0%; NPS samples had 7.4% positivity, ANS samples had 4.4% positivity, and saliva samples had 4.8% positivity. Among the 1,076 participants, 51.9% (559) were men, 57.1% (614) were >50 years of age, 81.2% (874) were Black (non-Hispanic), and 74.9% (806) had >1 chronic medical condition (Table 1). Most (80.0%; 861) participants were enrolled in the emergency department: nearly half sought care for a COVID-19–related concern (18.2%; 196) or had a chief complaint including COVID-19–like symptoms (30.6%; 329). Over half (56.7%; 610) of participants had >1 current COVID-19 symptom; among currently symptomatic participants, 68.9% (420) reported symptom onset <1 week previously.

**Table 1:** Characteristics of patients in study of diagnostic performance of self-collected samples for severe acute respiratory syndrome coronavirus 2 testing, Atlanta, Georgia, USA*

Characteristic	Total	PCR results for nasopharyngeal swab sample†	
Positive	Negative
Total	1,076 (100.0)	80 (100.0)	926 (100.0)
Sex			
F	514 (47.8)	36 (45.0)	445 (48.1)
M	559 (51.9)	44 (55.0)	479 (51.7)
Nonbinary	2 (0.2)	0	2 (0.2)
Refused/unknown	1 (0.1)	0	0
Age, y			
18–29	145 (13.5)	8 (10.0)	122 (13.2)
30–39	155 (14.4)	12 (15.0)	128 (13.8)
40–49	162 (15.1)	18 (22.5)	139 (15.0)
50–59	308 (28.6)	20 (25.0)	271 (29.3)
>60	306 (28.4)	22 (27.5)	266 (28.7)
Race/ethnicity			
American Indian/Alaska Native, non-Hispanic	2 (0.2)	0	2 (0.2)
Asian, non-Hispanic	10 (0.9)	1 (1.3)	9 (1.0)
Black, non-Hispanic	874 (81.2)	67 (83.8)	746 (80.6)
Hispanic/Latinx	71 (6.6)	8 (10.0)	60 (6.5)
Multiple race	16 (1.5)	0	15 (1.6)
Native Hawaiian/Pacific Islander, non-Hispanic	1 (0.1)	0	1 (0.1)
Unknown/other/refused	21 (2.0)	2 (2.5)	18 (1.9)
White, non-Hispanic	81 (7.5)	2 (2.5)	75 (8.1)
Chronic medical conditions			
No condition reported	270 (25.1)	24 (30.0)	229 (24.7)
>1 condition reported	806 (74.9)	56 (70.0)	697 (75.3)
Emphysema or chronic obstructive pulmonary disorder	90 (8.4)	5 (6.3)	77 (8.3)
Asthma	224 (20.8)	13 (16.3)	192 (20.7)
Other chronic lung disease	65 (6.0)	6 (7.5)	55 (5.9)
Diabetes mellitus (type I or II)	259 (24.1)	22 (27.5)	219 (23.7)
Hypertension (high blood pressure)	491 (45.6)	32 (40.0)	425 (45.9)
Chronic heart or cardiovascular disease	154 (14.3)	8 (10.0)	135 (14.6)
Chronic kidney disease	76 (7.1)	2 (2.5)	68 (7.3)
Liver disease	40 (3.7)	3 (3.8)	34 (3.7)
Immunocompromising conditions‡	91 (8.5)	6 (7.5)	78 (8.4)
Neurologic or neurodevelopmental disorders§	110 (10.2)	4 (5.0)	94 (10.2)
Cancer (including in remission)	99 (9.2)	2 (2.5)	91 (9.8)
Other chronic diseases	178 (16.5)	12 (15.0)	155 (16.7)
Body mass index¶			
Underweight	36 (3.4)	3 (3.8)	28 (3.0)
Normal weight	366 (34.0)	18 (22.5)	327 (35.3)
Overweight	271 (25.2)	18 (22.5)	236 (25.5)
Obese	394 (36.6)	40 (50.0)	328 (35.4)
Unknown	9 (0.8)	1 (1.2)	7 (0.8)
Enrollment location			
Emergency department	861 (80.0)	74 (92.5)	722 (78.0)
Labor and delivery department	15 (1.4)	0	13 (1.4)
Preoperative screening clinic	200 (18.6)	6 (7.5)	191 (20.6)
Reason for visit			
COVID-19 concern	196 (18.2)	31 (38.8)	153 (16.5)
No COVID-19 concern, but chief complaint included COVID-19–like symptoms	329 (30.6)	25 (31.2)	280 (30.2)
Admission to labor and delivery unit	10 (0.9)	0	8 (0.9)
Preoperative requirements	180 (16.7)	4 (5.0)	173 (18.7)
Other reasons	361 (33.6)	20 (25.0)	312 (33.7)
Known close contact with COVID-19 patient			
Yes	129 (12.0)	16 (20.0)	107 (11.6)
Yes, <14 d since most recent exposure	30 (2.8)	9 (11.3)	19 (2.1)
No	890 (82.7)	56 (70.0)	775 (83.7)
Unknown	57 (5.3)	8 (10.0)	44 (4.8)
Reported a previous positive COVID-19 test			
Yes	52 (4.8)	11 (13.8)	37 (4.0)
No	1,024 (95.2)	69 (86.2)	889 (96.0)
COVID-19 symptom status			
Always asymptomatic	383 (35.6)	10 (12.5)	353 (38.1)
Currently asymptomatic	83 (7.7)	7 (8.8)	70 (7.6)
Currently symptomatic	610 (56.7)	63 (78.7)	503 (54.3)
Days since symptom onset#			
0–2	187 (30.7)	19 (30.2)	150 (29.8)
3–7	233 (38.2)	26 (41.2)	194 (38.6)
8–14	84 (13.8)	12 (19.0)	67 (13.3)
>15	86 (14.1)	3 (4.8)	76 (15.1)
Unknown	20 (3.3)	3 (4.8)	16 (3.2)
Current signs and symptoms#			
Fever, measured	52 (8.5)	14 (22.2)	32 (6.4)
Fever, subjective	122 (20.0)	26 (41.3)	88 (17.5)
Cough	324 (53.1)	42 (66.7)	266 (52.9)
Shortness of breath or difficulty breathing	346 (56.7)	33 (52.4)	289 (57.5)
Fatigue	306 (50.2)	43 (68.3)	243 (48.3)
Muscle or body aches	275 (45.1)	34 (54.0)	224 (44.5)
Headaches	212 (34.8)	28 (44.4)	168 (33.4)
New loss of taste	101 (16.6)	20 (31.7)	71 (14.1)
New loss of smell	72 (11.8)	18 (28.6)	48 (9.5)
Sore throat	135 (22.1)	14 (22.2)	112 (22.2)
Congestion or runny nose	230 (37.7)	23 (36.5)	188 (37.4)
Nausea	150 (24.6)	20 (31.7)	120 (23.9)
Vomiting	73 (12.0)	6 (9.5)	63 (12.5)
Diarrhea	121 (19.8)	11 (17.5)	103 (20.5)
Current symptom groups#			
Respiratory symptoms	514 (84.3)	51 (81.0)	426 (84.7)
Upper respiratory symptoms	291 (47.7)	28 (44.4)	242 (48.1)
Lower respiratory symptoms	459 (75.2)	46 (73.0)	383 (76.1)
Nonrespiratory symptoms	504 (82.6)	58 (92.1)	411 (81.7)
Upper respiratory and loss of taste or smell	81 (13.3)	11 (17.5)	64 (12.7)
Gastrointestinal symptoms	214 (35.1)	23 (36.5)	178 (35.4)
Nonrespiratory symptoms excluding loss of taste or smell	493 (80.8)	55 (87.3)	404 (80.3)
Nonconstitutional symptoms	150 (24.6)	8 (12.7)	131 (26.0)
Case definitions#			
COVID-19–like symptoms	106 (17.4)	23 (36.5)	77 (15.3)
COVID-19**	553 (90.7)	58 (92.1)	455 (90.5)
Influenza-like illness	34 (5.6)	10 (15.9)	19 (3.8)
Acute respiratory infection††	513 (84.1)	50 (79.4)	426 (84.7)

*Values are no. (%). Percentages rounded to form a sum of 100.0%. COVID-19, coronavirus disease. †Using CDC 2019-nCoV Real-Time Reverse Transcriptase PCR Diagnostic Panel (*15*). ‡For example, HIV co-infection (not virally suppressed) or history of chemotherapy within past 12 mo, solid organ or bone marrow transplant, long-term steroid use (i.e., 20 mg for >1 mo), immunosuppressant use, or use of tumor necrosis factor alpha inhibitors. §e.g., seizure disorders, Alzheimer’s disease, dementia, traumatic brain injuries, or stroke. ¶Calculated using self-reported height and weight. #Among currently symptomatic participants. **According to definition established by the Council of State and Territorial Epidemiologists (*17*). ††According to definition established by the World Health Organization (*18*).

Most (93.5%; 1,006) participants provided an NPS sample, of which 8.0% (80/1,006) tested positive for SARS-CoV-2. A total of 911 participants had an RT-PCR result for all 3 specimens (i.e., saliva, ANS, and NPS samples), 10 participants had results for only saliva and NPS samples, and 84 participants had results for only ANS and NPS samples (Figure 1).

### Performance of Self-Collected Sample Types

Among 995 participants who provided ANS and NPS samples that produced definitive results, 963 (96.8%) had concordant results (κ = 0.73, 95% CI 0.64–0.82). Compared with NPS samples, ANS samples had 59% sensitivity (95% CI 47%–70%), 100% specificity (95% CI 100%–100%), 100% PPV (95% CI 92%–100%), and 97% NPV (95% CI 95%–98%). Among 921 participants who provided saliva and NPS samples that produced definitive results, 894 (97.1%) had concordant results (κ = 0.76, 95% CI 0.67–0.85). Compared with NPS samples, saliva had 68% sensitivity (95% CI 55%–78%), 99% specificity (95% CI 99%–100%), 90% PPV (95% CI 79%–97%), and 97% NPV (95% CI 96%–98%).

To understand the benefit of using both self-collected specimens for diagnosis, we analyzed data from 1,005 participants who had definitive results for >1 self-collected specimen. We found that 977 (97.2%) had concordant results between the self-collected combination and NPS samples (κ = 0.79, 95% CI 0.71–0.86). Using NPS as the comparator, we found self-collected combination samples had 71% sensitivity (95% CI 60%–81%), 99% specificity (95% CI 99%–100%), 92% PPV (95% CI 82%–97%), and 98% NPV (95% CI 96%–98%). When any positive was used as the comparator, we observed little change in the overall findings: the overall sensitivity of the ANS swab sample decreased slightly, the sensitivity of saliva samples increased slightly, and sensitivity of self-collected combination samples increased slightly (Appendix Table 1).

### Sensitivity by Patient Characteristics and Symptoms

Saliva and self-collected combination samples had higher overall sensitivities than ANS samples; this pattern was reflected among men, participants 18–29 years of age and 50–59 years of age, and Black (non-Hispanic) participants (Table 2). Among Hispanic/Latinx participants, sensitivity was significantly lower for saliva and self-collected combination samples. Sensitivity was higher among those not reporting any chronic medical conditions, whose reason for hospital visit was a COVID-19–related concern or whose chief complaint included COVID-19–like symptoms, who reported close contact to a COVID-19 patient during the previous <14 days, and who did not report a previous positive COVID-19 test. Sensitivity was lower among participants who reported a previous positive COVID-19 test (Table 2).

**Table 2:** Sensitivity of various self-collected sample types compared with HCW-collected samples in study of severe acute respiratory syndrome coronavirus 2 testing, by patient characteristic, Atlanta, Georgia, USA*

Characteristic	Sample sensitivity compared with HCW-collected samples, % (95% CI)†		
	Anterior nasal swab	Saliva	Self-collected combination‡
Total	59 (47–70)	68 (55–78)	71 (60–81)
Sex			
F	53 (35–70)	63 (44–80)	67 (49–81)
M	64 (48–78)	71 (54–85)	74 (59–86)
Age, y			
18–29	75 (35–97)	83 (36–100)	88 (47–100)
30–39	45 (17–77)	44 (14–79)	45 (17–77)
40–49	47 (23–72)	65 (38–86)	67 (41–87)
50–59	70 (46–88)	89 (65–99)	85 (62–97)
>60	59 (36–79)	56 (31–78)	68 (45–86)
Race/ethnicity			
Black, non-Hispanic	63 (50–75)	72 (59–83)	76 (64–85)
Hispanic/Latinx	25 (3–65)	**25 (3–65)**	**25 (3–65)**
White, non-Hispanic	100 (16–100)	100 (2–100)	100 (16–100)
Chronic medical conditions			
0	75 (51–91)	83 (59–96)	81 (58–95)
>1	54 (40–67)	62 (47–76)	68 (54–80)
Body mass index§			
Underweight	33 (1–91)	0 (0–84)	33 (1–91)
Normal weight	71 (44–90)	67 (38–88)	72 (47–90)
Overweight	59 (33–82)	67 (38–88)	71 (44–90)
Obese	57 (41–73)	74 (57–88)	75 (59–87)
Reason for visit			
COVID-19 concern	63 (44–80)	73 (52–88)	77 (58–90)
No COVID-19 concern, but chief complaint included COVID-19–like symptoms	71 (49–87)	81 (58–95)	80 (59–93)
Preoperative requirements or admission to labor and delivery unit	25 (1–81)	25 (1–81)	25 (1–81)
Other reasons	45 (23–68)	53 (28–77)	60 (36–81)
Known close contact			
Yes	56 (30–80)	67 (35–90)	69 (41–89)
Yes, <14 d since most recent exposure	67 (30–93)	80 (28–99)	78 (40–97)
No	60 (46–73)	67 (52–80)	71 (57–82)
Unknown	57 (18–90)	71 (29–96)	75 (35–97)
Reported a previous positive COVID-19 test			
Yes	**9 (0–41)**	**33 (7–70)**	**27 (6–61)**
No	67 (55–78)	73 (60–84)	78 (66–87)
COVID-19 symptom status			
Always asymptomatic	40 (12–74)	44 (14–79)	50 (19–81)
Currently asymptomatic	57 (18–90)	67 (22–96)	57 (18–90)
Currently symptomatic	62 (49–74)	72 (58–83)	76 (63–86)
Days since symptom onset¶			
0–2	72 (47–90)	71 (44–90)	78 (52–94)
3–7	**80 (59–93)**	85 (62–97)	**88 (70–98)**
8–14	33 (10–65)	73 (39–94)	67 (35–90)
>15	0 (0–71)	33 (1–91)	33 (1–91)
Current individual symptoms¶			
Fever, measured	**93 (66–100)**	82 (48–98)	93 (66–100)
Fever, subjective	68 (46–85)	76 (53–92)	80 (59–93)
Cough	66 (49–80)	81 (64–92)	83 (69–93)
Shortness of breath or difficulty breathing	59 (41–76)	72 (53–87)	76 (58–89)
Fatigue	62 (46–76)	76 (59–88)	77 (61–88)
Muscle or body aches	67 (48–82)	77 (58–90)	79 (62–91)
Headaches	63 (42–81)	69 (48–86)	71 (51–87)
New loss of taste	75 (51–91)	71 (44–90)	75 (51–91)
New loss of smell	72 (47–90)	81 (54–96)	83 (59–96)
Sore throat	57 (29–82)	62 (32–86)	64 (35–87)
Congestion or runny nose	74 (52–90)	81 (58–95)	83 (61–95)
Nausea	58 (33–80)	74 (49–91)	75 (51–91)
Vomiting	60 (15–95)	67 (22–96)	67 (22–96)
Diarrhea	45 (17–77)	80 (44–97)	82 (48–98)
Current symptom groups¶			
Respiratory symptoms	64 (49–77)	74 (59–86)	78 (65–89)
Upper respiratory symptoms	64 (44–81)	73 (52–88)	75 (55–89)
Lower respiratory symptoms	64 (49–78)	76 (60–89)	80 (66–91)
Nonrespiratory symptoms	62 (49–75)	73 (59–85)	77 (64–87)
Upper respiratory and loss of taste or smell	73 (39–94)	70 (35–93)	73 (39–94)
Gastrointestinal symptoms	55 (32–76)	77 (55–92)	78 (56–93)
Nonrespiratory symptoms excluding loss of taste or smell	64 (50–77)	74 (59–86)	78 (64–88)
Nonconstitutional symptoms	50 (16–84)	57 (18–90)	62 (24–91)
Common case definitions¶			
COVID-19–like symptoms	70 (47–87)	79 (54–94)	83 (61–95)
COVID-19#	62 (49–75)	73 (59–85)	77 (64–87)
Influenza-like illness	**90 (55–100)**	88 (47–100)	90 (55–100)
Acute respiratory infection**	63 (48–77)	74 (59–86)	78 (64–88)

*HCW-collected samples were nasopharyngeal. Boldface type indicates values that are significantly different (p<0.05) than overall sensitivity values, according to a 1-sample test of proportions. COVID-19, coronavirus disease; HCW, healthcare worker. †Using CDC 2019-nCoV Real-Time Reverse Transcriptase PCR Diagnostic Panel (*15*). ‡Self-collected combination reflects >1 positive result in a patient’s paired anterior nasal swab and saliva samples. §Calculated using self-reported height and weight. ¶Among currently symptomatic participants. #According to definition established by the Council of State and Territorial Epidemiologists (*17*). **According to definition established by the World Health Organization (*18*).

Sensitivity was higher among samples from currently symptomatic participants (62% for ANS, 72% for saliva, and 76% for self-collected combination) and was highest among samples from participants who provided samples 3–7 days after symptom onset (80% for ANS, 85% for saliva, and 88% for self-collected combination); these differences were statistically significant for ANS and self-collected combination samples (p<0.05). Sensitivity was higher for most individual symptoms, but highest among participants reporting measured fever, congestion or runny nose, new loss of smell, new loss of taste, cough, or subjective fever. Similarly, sensitivity was higher among participants who met most symptom case definitions, but highest among patients who had influenza-like illness, COVID-19–like symptoms, or upper respiratory symptoms accompanied by loss of smell or taste. Sensitivity was lower among patients with nonconstitutional symptoms (Table 2).

### C_t_ Values

**Figure 2:**
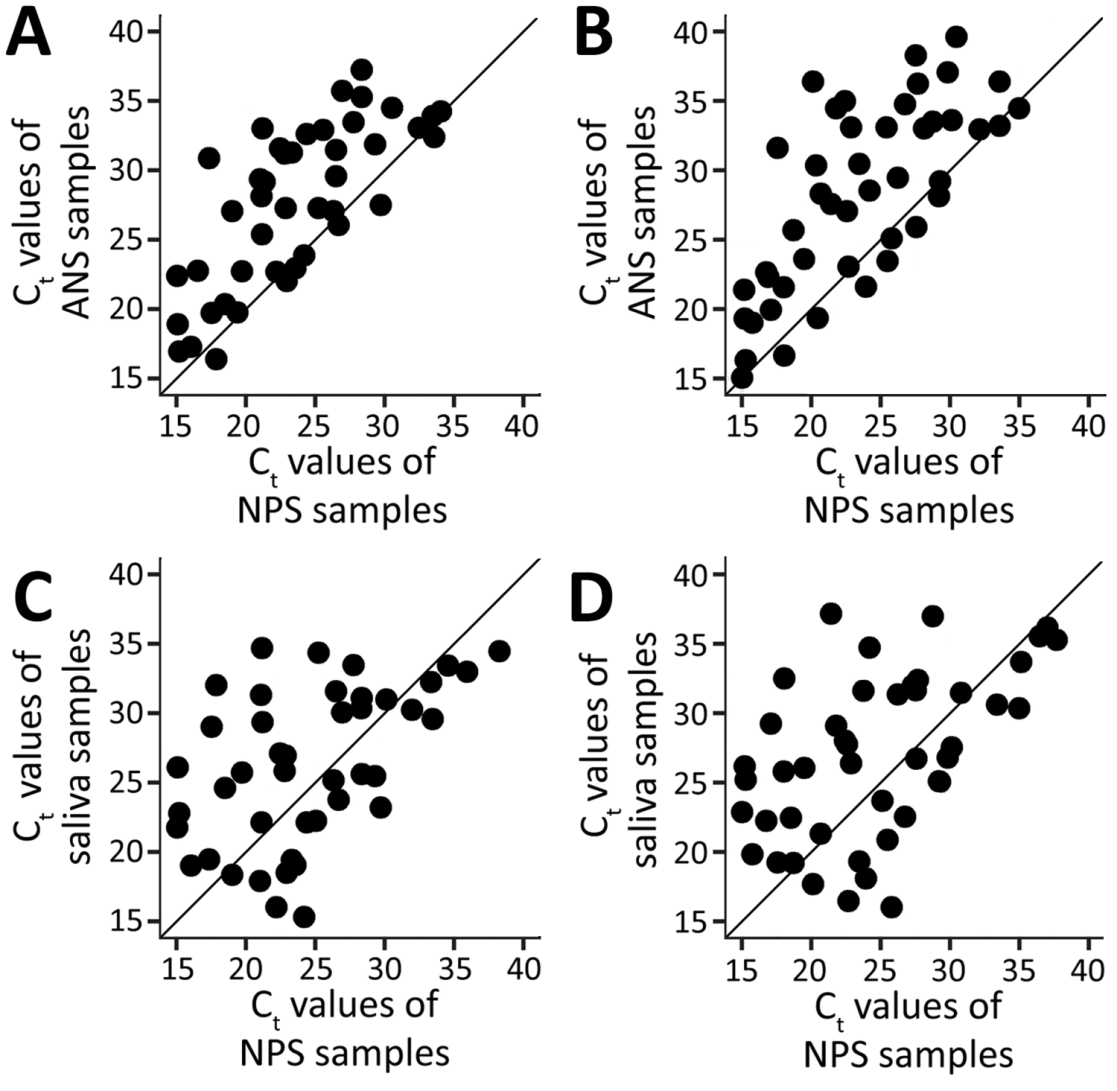
C_t_ values of self-collected and healthcare worker–collected samples for severe acute respiratory syndrome coronavirus 2 testing, Atlanta, Georgia, USA. PCR completed using CDC 2019-nCoV Real-Time Reverse Transcriptase PCR Diagnostic Panel (*15*). A) ANS and NPS samples at PCR target N1. B) ANS and NPS samples at PCR target N2. C) NPS and saliva samples at PCR target N1. D) NPS and saliva samples at PCR target N2. ANS, anterior nasal swab; C_t_, cycle threshold; NPS, nasopharyngeal swab.

**Figure 3:**
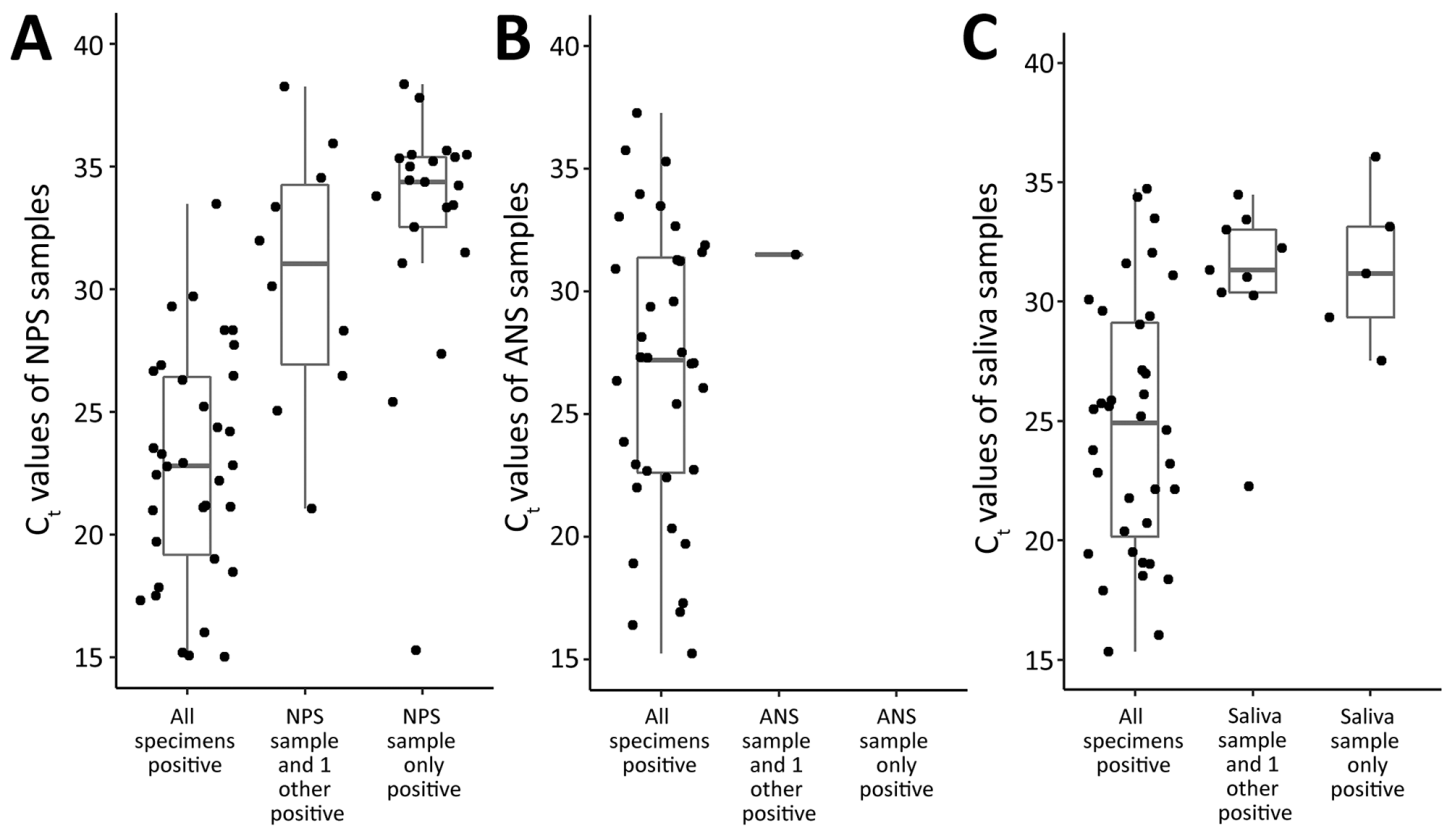
C_t_ values of self-collected and healthcare worker–collected samples for N1 target of severe acute respiratory syndrome coronavirus 2 PCR, Atlanta, Georgia, USA. PCR completed using CDC 2019-nCoV Real-Time Reverse Transcriptase PCR Diagnostic Panel (*15*). Horizontal lines within boxes indicate medians; box tops and bottoms indicate 25th and 75th percentiles; whiskers indicate the range. ANS, anterior nasal swab; C_t_, cycle threshold; NPS, nasopharyngeal swab.

Among 46 participants with positive ANS and NPS samples, 85% (for PCR target N1) and 78% (for PCR target N2) had an ANS sample with a higher C_t_ value than that of its paired NPS sample (Figure 2, panels A, B). We observed a moderate positive correlation between the C_t_ values of ANS and NPS samples (r = 0.75 for N1, r = 0.71 for N2); both targets had median NPS C_t_ values of 22.8 (range 14.6–34.1 for N1, 14.7–35.0 for N2). Among 46 participants with positive saliva and NPS samples, 57% (N1) and 59% (N2) had a saliva sample with a higher C_t_ value than that of its paired NPS sample (Figure 2, panels C, D). We observed a low positive correlation between the C_t_ values of saliva and NPS samples (r = 0.53 for both N1 and N2); targets had median NPS C_t_ values of 23.1 (range 14.6–38.3) for N1 and 23.8 (range 14.7–37.7) for N2. When limiting the analysis to the 72 participants who had 3 definitive and >1 positive result, C_t_ values were lowest when all paired specimens were SARS-CoV-2–positive; for N1, the median C_t_ values were 27.2 for ANS, 24.9 for saliva, and 22.6 for NPS samples (Figure 3). When <2 specimens were positive, all specimens had median C_t_ values >30. Participants who did not have COVID-19 symptoms had higher median NPS C_t_ values (33.5 for N1, 34.4 for N2) than did those who reported >1 COVID-19 symptom (25.6 for N1, p = 0.03; 26.9 for N2, p = 0.03). Among those reporting COVID-19 symptoms, participants who had symptom onset <1 week before testing had the lowest median NPS C_t_ values (23.5 vs. 30.8 for N1, p<0.01; 24.2 vs. 33.3 for N2, p<0.01).

## Discussion

In this investigation, we found that self-collected saliva samples had a higher sensitivity than self-collected ANS samples (68% vs. 59%) compared with HCW-collected NPS samples. However, each sample type had lower sensitivity than suggested by most previously published data (*3*,*6*,*19*–*21*). The self-collected combination had a higher sensitivity (71%) than NPS samples. We found that the sensitivity of self-collected samples (separately and in combination) differed according to patient characteristics. The presence of COVID-19 symptoms at time of specimen collection and the time since symptom onset affected sensitivity. We also noted differences in sensitivity across demographic groups, possibly reflecting differences in access to care or care-seeking behavior rather than differences in viral shedding. Our results illustrate that certain patient characteristics are associated with the sensitivity of self-collected specimens used for RT-PCR.

We found lower sensitivities for saliva and ANS samples than those for most other published studies, including 2 recent meta-analyses that found saliva samples to have sensitivities of 83.2% and 86.9% (*21*,*22*). Many studies showing high sensitivity of self-collected specimens enrolled symptomatic patients who were recently hospitalized for confirmed COVID-19 or whose symptom onset was <1 week before sample collection (*3*,*4*). A strength of our investigation was that we included symptomatic and asymptomatic patients being tested for SARS-CoV-2 for screening and diagnostic purposes. Because a substantial proportion of patients infected with SARS-CoV-2 have asymptomatic or mild illness, physicians must be able to analyze results in the context of test sensitivity in patients with few or no symptoms. Sample sensitivity was highest among participants reporting symptom onset 3–7 days before sample collection. Similarly, another study found that the sensitivity of saliva samples was highest (95%) among symptomatic patients tested <1 week after symptom onset and lowest (50%) among patients tested >1 week after symptom onset (*23*). The sample sensitivity differences among patients with different demographic characteristics might reflect differences in access to care or healthcare-seeking behavior. Delayed access to care might postpone specimen collection, decreasing the sensitivity of the samples. For example, Hispanic/Latinx participants who had a positive NPS sample had longer symptom duration compared with participants of other race/ethnicity categories (data not shown).

Using 2 self-collected specimens could increase overall test sensitivity, which reached 88% among participants whose symptoms began 3–7 days before sample collection. Similarly, Tan et al. (*24*) found that combining self-collected oropharynx and midturbinate swab and saliva results increased test sensitivity. Using multiple noninvasive specimens might improve SARS-CoV-2 detection in persons tested <1 week after symptom onset and reduce demand for PPE and HCW exposure. However, testing multiple specimens might put additional strain on laboratory systems that are already overburdened. Pooling self-collected specimens before testing might alleviate some of this additional strain on laboratories, but this practice should be investigated further for accuracy.

Similar to other studies, we found that most NPS samples had lower C_t_ values than did their paired saliva and ANS samples (*4*,*25*,*26*). We also found that median C_t_ values were lowest when all 3 specimens were SARS-CoV-2–positive; the median C_t_ value increased to >30 when <2 specimens were positive (Figure 3). The lower sensitivity in this investigation might be due to high C_t_ value discordant specimens, which can occur as infection subsides. We also found slightly higher overall median C_t_ values for NPS samples than reported in similar studies (*5*,*27*). However, many of these previous studies were implemented earlier in the pandemic when previous infection or exposure was less common. Our investigation began after the first 2 peaks in Atlanta; by the end of enrollment, Atlanta was entering its third peak. When the C_t_ value of the NPS sample is high, discordance with the self-collected specimens also could increase. Salvatore et al. (*28*) found that C_t_ values for NPS samples were lowest <1 week after symptom onset. Furthermore, Wolfel et al. (*29*) found viral subgenomic mRNA in throat swab specimens collected <5 days after symptom onset and in sputum samples taken 4–11 days after symptom onset, indicating active infection. Although C_t_ values are not directly correlated with viral load, they provide a semiquantitative assessment of viral RNA concentration.

Specimen collection method might also affect sensitivity. Procop et al. (*5*) compared NPS and enhanced saliva samples (i.e., self-collected nasal secretions or mucus, phlegm, and saliva stored in a single tube) and found 100% positive agreement and 99.4% negative agreement. This method of saliva collection provides a mixture of upper and lower respiratory secretions, thereby enabling detection for a longer time after symptom onset. We used general spitting for saliva collection, which has the lowest sensitivity estimate in comparison with other saliva collection methods (*22*). General spitting does not require special devices or transport media, enabling our methods and results to be broadly applicable. The duration and pressure applied while swabbing the anterior nares also might affect ANS sample quality. The sequence of specimen collection, which was not always clear from published studies, could also affect sensitivity (*19*). In our investigation, saliva and ANS samples were collected before NPS samples. Collecting ANS samples after NPS samples could displace virus from nasopharyngeal tissue or midturbinate before the swab leaves the nares, thereby biasing self-collected ANS toward higher sensitivity.

The first limitation of our study is that because we used a cross-sectional design, we did not have information on whether patients were asymptomatic or presymptomatic at specimen collection; we also did not have data on disease severity. In presymptomatic patients, samples might have been collected too early to detect viral RNA in some or all specimen types. Second, we lacked the statistical power to detect significant differences in sensitivity by most variables because the initial sample size calculation assumed self-collected specimens to have >90% sensitivity. Third, all responses were self-reported and could have been affected by recall bias. Fourth, the CDC 2019-nCoV rRT-PCR Diagnostic Panel has a higher limit of detection than many commercially available, high-throughput assays (*30*), limiting our ability to detect lower concentrations of viral RNA. However, the clinical and public health utility of detecting these lower concentrations is unknown. Fifth, the CDC 2019-nCoV rRT-PCR Diagnostic Panel does not currently include saliva; instead, the assay was designed for qualitative detection of nucleic acid from SARS-CoV-2. Of 1,006 NPS samples, 64 aliquots did not meet storage requirements approved under the assay’s instructions for use (samples should be stored at 4°C for <72 hours after collection) (*15*). Because some samples were stored for longer than recommended, viral RNA degradation might have affected the assay’s performance. Because the CDC RT-PCR results were not used for clinical care, excluding these specimens did not change sensitivity; furthermore, CDC RT-PCR results were in concordance with the hospital’s RT-PCR results (data not shown). As a result, we decided to include these specimens in the analysis (Appendix). Our findings might not be generalizable to other assays or techniques. Sixth, heterogenous self-collection coaching techniques might have introduced differences in the quality of samples collected under the guidance of different interviewers. Finally, PCR does not indicate whether active replication is occurring. Therefore, we are unable to determine whether patients with positive NPS samples but negative saliva or ANS samples have older infections or if the self-collected specimens are less sensitive than NPS samples. Additional laboratory testing is required to clarify the viability of different specimen types and how viability affects clinical presentation and transmissibility. Our study highlights that the sensitivities of saliva and ANS samples are different than that of NPS samples. These findings show that physicians should consider the patient’s clinical history, exposures, and time of symptom onset when interpreting PCR results.

Overall, the sensitivities of ANS and saliva samples were lower than that of NPS samples from patients being tested for SARS-CoV-2 for diagnostic and screening purposes. The sample sensitivity was highest among participants with symptom onset within 3–7 days of specimen collection, especially when the reason for the patient visit was COVID-19–related, and those not reporting a previous positive test. Encouraging persons to seek testing within a week of symptom onset could increase the accuracy and usefulness of self-collected specimens used for diagnosing SARS-CoV-2 infections. It is important that clinicians are aware of how differences in patient characteristics and specimen type can affect test sensitivity. Testing programs and clinical settings might consider patient characteristics, previous test results, and timing of symptom onset when determining which specimen types to use.

Appendix Additional information on the effects of patient characteristics on diagnostic performance of self-collected samples for SARS-CoV-2 testing.
