# Supplementary material for: Effects of Patient Characteristics on Diagnostic Performance of Self-Collected Samples for SARS-CoV-2 Testing
Source: Emerg Infect Dis. 2021 Aug;27(8):2081–9. doi: 10.3201/eid2708.210667 (PMC8314823; doi:10.3201/eid2708.210667)
Supplement: Appendix — Additional information on the effects of patient characteristics on diagnostic performance of self-collected samples for SARS-CoV-2 testing. [file 21-0667-Techapp-s1.pdf]

Article DOI: https://doi.org/10.3201/eid2708.210667

Effects of Patient Characteristics on Performance of Self-Collected Samples for SARS-CoV-2 Testing

Appendix

Appendix Methods

Specimen Collection

Patients collected ≈2 mL raw saliva into a sterile 50 mL conical centrifuge tube. Patients were then advised to collect anterior nasal swab (ANS) samples by inserting a mini flocked tip swab into 1 anterior naris, twirling the swab for 10 s, removing the swab and placing it directly into the other naris, and twirling again for 10 s. Upon collection, ANS samples were stored in 3 mL universal viral transport media (Becton, Dickinson and Company, https://www.bd.com). All self-collected specimens were stored at 4°C or frozen (ANS samples only) before extraction. After interview and self-collection of specimens were completed, a nasopharyngeal swab (NPS) sample was collected by healthcare workers at Grady Memorial Hospital (Atlanta, Georgia, USA) using flocked foam swabs and placed into a vial of 3 mL Smart Transport Medium (MedSchenker, https://medschenker.com). Reverse transcription PCR (RT-PCR) on the NPS samples was performed the same day by the hospital laboratory and these results were used for clinical care per hospital protocol and not included in the diagnostic performance analysis. NPS samples were stored at 4°C and remnant Smart Transport Medium was aliquoted and transferred to the US Centers for Disease Control and Prevention (CDC; Atlanta) laboratory for RT-PCR.

Extraction and Testing Methods

Nucleic acid was extracted from 120 μL of viral transport media (ANS and NPS samples) or 120 μL whole saliva at CDC by using the Maxwell RSC 48 automated extraction platform and Maxwell RSC Viral Total Nucleic Acid Purification Kit (Promega, https://www.promega.com). Preprocessing was performed to liquify high-viscosity saliva specimens before extraction by using equal volumes of whole saliva and dithiothreitol and incubated for 30 min at room

temperature. RNA was eluted into 75 μL of nuclease-free water and a 5 μL sample was tested by using the CDC 2019-nCoV Real-Time Reverse transcriptase PCR Diagnostic Panel according to the emergency use authorization instructions for use (IFU) (*1*,*2*). Samples with amplification (cycle threshold <40) of both N1 and N2 gene targets were considered positive, amplification of only 1 N gene target was inconclusive, and amplification of neither N gene target was negative. Lower cycle threshold values represent higher viral RNA concentration. Some NPS aliquots from the hospital were stored outside of the IFU requirements (samples were stored at 4°C for >72 h after collection). Although stability of severe acute respiratory syndrome coronavirus 2 (SARS-CoV-2) RNA has been demonstrated at 4°C in universal viral transport media for up to 14 days for other RT-PCR assays (*3*,*4*), an analysis excluding NPS aliquots stored outside of IFU requirements was performed to ensure storage conditions did not influence the findings.

Symptom Group and Clinical Case Definitions

Respiratory symptoms were defined as any of the following: runny nose/congestion, sore throat (upper respiratory); or cough, shortness of breath/difficulty breathing, wheezing, and chest pain (lower respiratory). Nonrespiratory symptoms were defined as any of the following: fever (measured or subjective), muscle/body aches, headache, chills, fatigue, loss of smell, loss of taste, nausea, vomiting, and diarrhea. COVID-19–like symptoms were defined as fever (measured or subjective) and cough or shortness of breath/difficulty breathing (*5*). Council of State and Territorial Epidemiologists clinical criteria for COVID-19 reporting was defined as any of the following: cough, shortness of breath/difficulty breathing, new smell disorder, or new taste disorder or ≥2 of the following: fever (measured or subjective), chills, myalgia, headache, sore throat, nausea or vomiting, diarrhea, fatigue, or congestion/runny nose (*6*). Influenza-like illness was defined as measured fever and cough or sore throat (*5*). The World Health Organization defines acute respiratory infection as any shortness of breath/difficulty breathing, cough, sore throat, or congestion/runny nose (*7*).

Appendix Results

Storage Requirement Analysis

Among 1,006 patients with NPS RT-PCR results, 64 NPS samples did not meet the IFU storage requirement of 4°C for ≤72 h from collection. These 64 specimens were received and

tested a median of 4 d after collection (range 4–5 d). The overall sensitivity of ANS samples compared with NPS samples was 59% (95% CI 47%–70%); excluding the specimens that did not meet storage requirements slightly changed the sensitivity (58%; 95% CI 46%–69%). The overall sensitivity of saliva samples compared with NPS samples was 68% (95% CI 55%–78%); excluding the specimens that did not meet storage requirements slightly changed the sensitivity point estimate (67%; 95% CI 54–78). There was no change in specificity. Among those with an RT-PCR result from Grady Memorial Hospital (n = 983), there was no difference in NPS result concordance of CDC RT-PCR results with Grady RT-PCR results among those that did not meet the storage requirement and those that did meet the storage requirement (61/62, 98.4% vs. 907/921, 98.5%; p = 1.0 by Fisher exact test).

Sensitivities of Various Sample Types Compared with Any Positive Result

Because NPS samples do not detect all SARS-CoV-2 infections, we reran the sensitivity analysis with a combined variable for any positive result from ANS, saliva, or NPS samples as the comparator. We calculated sensitivity and 95% CI of self-collected specimens within patient characteristic subgroups to evaluate how patient differences might affect specimen performance. We determined statistically significant sensitivity differences between overall and subgroups using a 1-sample, 2-way test of proportions ($p<0.05$). Among 1,076 per-protocol patients, 85 (7.9%) had ≥1 ANS, saliva, or NPS sample test positive for SARS-CoV-2. Among 1,063 participants with a definitive ANS sample result, ANS sample sensitivity compared with any positive was 56% (95% CI 45–67) (Appendix Table). Compared with the ANS versus NPS sample analysis, the only change in statistical significance was the sensitivity among those reporting symptom onset 3–7 d before enrollment was not significantly different than the overall sensitivity. Among 989 participants with a definitive saliva sample result, sensitivity of saliva compared to any positive result was 70% (95% CI 59–80). Compared with the saliva versus NPS sample analysis, the only change in statistical significance was the sensitivity among those with a previous positive result was not significantly different than the overall sensitivity. Among 1,075 participants with a definitive ANS or saliva sample result, sensitivity of self-collected combination compared to any positive was 73% (95% CI 62–82). Compared with the self-collected combination versus NPS sample analysis, the only change in statistical significance was the sensitivity among those reporting symptom onset 3–7 d before enrollment was not significantly different than the overall sensitivity.

References

1. Lu X, Wang L, Sakthivel SK, Whitaker B, Murray J, Kamili S, et al. US CDC real-time reverse transcription PCR panel for detection of severe acute respiratory syndrome coronavirus 2. Emerg Infect Dis. 2020;26:1654–65. PubMed https://doi.org/10.3201/eid2608.201246

2. United States Food and Drug Administration. Emergency use authorization: CDC 2019-nCoV Real-Time RT-PCR Diagnostic Panel. 2020 [cited 2021 Mar 2]. https://www.fda.gov/media/134919/download

3. Rogers AA, Baumann RE, Borillo GA, Kagan RM, Batterman HJ, Galdzicka MM, et al. Evaluation of transport media and specimen transport conditions for the detection of SARS-CoV-2 by use of real-time reverse transcription-PCR. J Clin Microbiol. 2020;58:e00708–20. PubMed https://doi.org/10.1128/JCM.00708-20

4. Kim N, Kwon A, Roh EY, Yoon JH, Han MS, Park S-W, et al. Effects of storage temperature and media/buffer for SARS-CoV-2 nucleic acid detection. Am J Clin Pathol. 2021;155:280–5. PubMed https://doi.org/10.1093/ajcp/aqaa207

5. Boehmer TK, DeVies J, Caruso E, van Santen KL, Tang S, Black CL, et al. Changing age distribution of the COVID-19 pandemic—United States, May–August 2020. MMWR Morb Mortal Wkly Rep. 2020;69:1404–1409.

6. Council of State and Territorial Epidemiologists. Update to the standardized surveillance case definition and national notification for 2019 novel coronavirus disease (COVID-19). 2020 [cited 2021 Jan 29]. https://cdn.ymaws.com/www.cste.org/resource/resmgr/ps/positionstatement2020/Interim-20-ID-02_COVID-19.pdf

7. World Health Organization. RSV surveillance case definitions. 2020 [cited 2021 January 29]. https://www.who.int/influenza/rsv/rsv_case_definition/en

Appendix Table. Sensitivity of self-collected samples for severe acute respiratory syndrome coronavirus 2 testing*

Characteristic	Sensitivity, % (95% CI)†		
	Anterior nasal swab sample	Saliva sample	Self-collected combination‡
Total	56 (45–67)	70 (59–80)	73 (62–82)
Sex			
F	50 (34–66)	68 (49–83)	70 (53–83)
M	61 (45–76)	72 (56–85)	76 (60–87)
Age, y			
18–29	67 (30–93)	86 (42–100)	89 (52–100)
30–39	43 (18–71)	58 (28–85)	57 (29–82)
40–49	44 (22–69)	67 (41–87)	68 (43–87)
50–59	67 (43–85)	89 (67–99)	86 (64–97)
>60	59 (36–79)	56 (31–78)	68 (45–86)
Race/ethnicity			
Black, non-Hispanic	60 (48–72)	75 (62–85)	77 (66–87)
Hispanic/Latino	25 (3–65)	**25 (3–65)**	**25 (3–65)**
White, non-Hispanic	67 (9–99)	100 (16–100)	100 (29–100)
Chronic medical conditions			
0	73 (50–89)	85 (62–97)	83 (61–95)
>1	51 (37–64)	65 (50–78)	69 (56–81)
Body mass index§			
Underweight	33 (1–91)	0 (0–84)	33 (1–91)
Normal weight	67 (41–87)	69 (41–89)	74 (49–91)
Overweight	56 (31–78)	69 (41–89)	72 (47–99)
Obese	55 (39–70)	77 (61–89)	77 (62–89)
Reason for visit			
COVID-19 concern	59 (41–75)	77 (58–90)	79 (62–91)
No COVID-19 concern, but chief complaint included COVID-19–like symptoms	68 (46–85)	82 (60–95)	81 (61–93)
Preoperative requirements or admission to labor and delivery unit	25 (1–81)	25 (1–81)	25 (1–81)
Other reasons	43 (22–66)	56 (31–78)	62 (38–82)
Known close contact			
Yes	53 (29–76)	73 (45–92)	74 (49–91)
Yes, <14 d since most recent exposure	70 (35–93)	83 (36–100)	80 (44–97)
No	58 (44–71)	69 (54–81)	72 (58–83)
Unknown	50 (16–84)	75 (35–97)	78 (40–97)
Reported a previous positive COVID-19 test			
Yes	**7 (0–34)**	50 (21–79)	**43 (18–71)**
No	66 (53–77)	74 (62–84)	79 (68–88)
COVID-19 symptom status			
Always asymptomatic	36 (11–69)	50 (19–81)	55 (23–83)
Currently asymptomatic	50 (16–84)	71 (29–96)	62 (24–91)
Currently symptomatic	60 (47–72)	74 (60–84)	77 (65–87)
Days since symptom onset¶			
0–2	74 (49–91)	72 (47–90)	79 (54–94)
3–7	74 (54–89)	86 (65–97)	89 (72–98)
8–14	33 (10–65)	73 (39–94)	67 (35–90)
>15	0 (0–60)	50 (7–93)	50 (7–93)
Current individual symptoms¶			
Fever, measured	**93 (68–100)**	83 (52–98)	93 (68–100)
Fever, subjective	69 (48–86)	77 (55–92)	81 (61–93)
Cough	62 (47–76)	82 (67–93)	85 (71–94)
Shortness of breath or difficulty breathing	56 (38–72)	76 (58–89)	78 (62–90)
Fatigue	60 (44–74)	78 (62–89)	78 (64–89)
Muscle or body aches	64 (46–79)	79 (61–91)	81 (65–92)
Headaches	60 (41–77)	72 (53–87)	74 (55–88)
New loss of taste	73 (50–89)	74 (49–91)	77 (55–92)
New loss of smell	70 (46–88)	83 (59–96)	85 (62–97)
Sore throat	50 (25–75)	67 (38–88)	69 (41–89)
Congestion or runny nose	72 (51–88)	83 (61–95)	84 (64–95)
Nausea	57 (34–78)	76 (53–92)	77 (55–92)
Vomiting	60 (15–95)	67 (22–96)	67 (22–96)
Diarrhea	46 (19–75)	83 (52–98)	85 (55–98)
Current symptom groups¶			
Respiratory symptoms	61 (47–74)	77 (62–88)	80 (67–90)

Characteristic	Sensitivity, % (95% CI)† Anterior nasal swab sample	Saliva sample	Self-collected combination‡
Upper respiratory symptoms	61 (42–78)	75 (56–90)	77 (59–90)
Lower respiratory symptoms	61 (46–75)	79 (63–90)	82 (69–91)
Nonrespiratory symptoms	61 (47–73)	75 (61–86)	78 (66–88)
Upper respiratory and loss of taste or smell	69 (39–91)	75 (43–95)	77 (46–95)
Gastrointestinal symptoms	54 (33–74)	79 (58–93)	80 (59–93)
Nonrespiratory symptoms excluding loss of taste or smell	62 (49–75)	76 (61–87)	79 (66, 89)
Nonconstitutional symptoms	44 (14–79)	62 (24–91)	67 (30–93)
Common case definitions¶			
COVID-19–like symptoms	71 (49–87)	80 (56–94)	83 (63–95)
COVID-19#	60 (47–72)	75 (62–86)	79 (66–88)
Influenza-like illness	**91 (59–100)**	89 (52–100)	91 (59–100)
Acute respiratory infection**	60 (46–74)	77 (62–88)	80 (66–89)

*COVID-19, coronavirus disease. Boldface type indicates values that are significantly different (p<0.05) than overall sensitivity values, according to a 1-sample test of proportions.
†Using CDC 2019-nCoV Real-Time Reverse Transcriptase PCR Diagnostic Panel (*2*). Results in comparison with all positive samples. Anterior nasal swab and saliva samples were self-collected; nasopharyngeal samples were collected by healthcare workers.
‡Self-collected combination reflects ≥1 positive result in a patient's paired anterior nasal swab and saliva samples.
§Calculated using self-reported height and weight.
¶Among currently symptomatic participants.
#According to definition established by the Council of State and Territorial Epidemiologists (*6*).
**According to definition established by the World Health Organization (*7*).

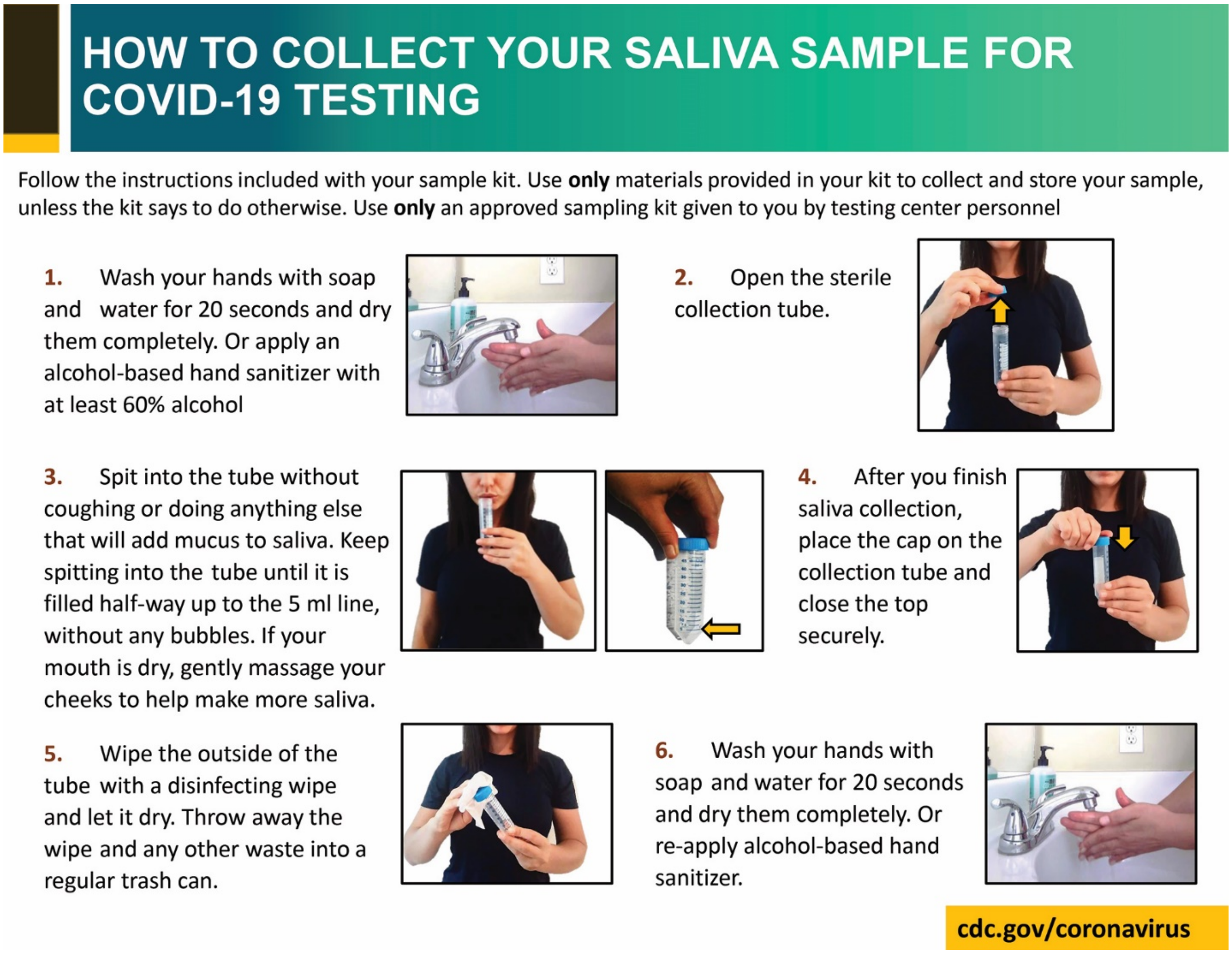

Appendix Figure. Infographic outlining steps for self-collection of saliva samples. Graphic provided to participants in study on sensitivity of self-collected samples for severe acute respiratory syndrome coronavirus 2 testing. COVID-19, coronavirus disease.
